# Molecular epidemiology and temporal evolution of norovirus associated with acute gastroenteritis in Amazonas state, Brazil

**DOI:** 10.1186/s12879-018-3068-y

**Published:** 2018-04-02

**Authors:** Juliana Merces Hernandez, Luciana Damascena Silva, Edivaldo Costa Sousa Junior, Renato Silva Bandeira, Elmer Abraão Martins Rodrigues, Maria Silvia Souza Lucena, Samya Thalita Picanço Costa, Yvone Benchimol Gabbay

**Affiliations:** 1 000 0004 0620 4442 Postgraduate Program in Virology, Evandro Chagas Institute, Rodovia BR-316, Km 7 s/n, Levilândia, Ananindeua, Pará 67030-000 Brazil; 2 000 0004 0620 4442 Virology Section, Evandro Chagas Institute, Brazilian Ministry of Health, Rodovia BR-316, Km 7 s/n, Levilândia, Ananindeua, Pará 67030-000 Brazil

**Keywords:** Norovirus, Acute gastroenteritis, Phylogenetic, Genetic diversity

## Abstract

**Background:**

Globally, Norovirus (NoV) is considered the most common cause of diarrheal episodes across all age groups. Despite its wide genetic diversity, the GII.4 strain is the most predominant and has been associated with epidemics worldwide. In this study, we characterized sporadic cases of diarrhea from NoV-positive children, during a five-year period (2010–2014).

**Methods:**

A total of 250 NoV-positive samples identified by an enzyme immunoassay (EIA) were subjected to RT-PCR and partial nucleotide sequencing for polymerase and capsid genes. Phylogenetic analysis was performed to identify NoV genotypes using the binary classification. In addition, sequences from the P2 subdomain (capsid) gene of GII-4 variants were characterized by evolutionary analyses, using the MCMC method implemented in the BEAST package. A 3D structure was built using protein modeling.

**Results:**

Phylogenetic analysis demonstrated a predominance of genotype GII.4 (52.4% - 99/189), variants New Orleans_2009 and Sydney_2012 followed by GII.P7/GII.6 with 6.3% (12/189). Amino acid analyses of the GII.4 strains showed several important amino acid changes. A higher evolutionary rate was found, 7.7 × 10^− 3^ in the Sydney variant and 6.3 × 10^− 3^ in the New Orleans. Based in evolutionary analysis the time to the most recent common ancestor (TMRCA) has been calculated as estimates of the population divergence time. Thus, TMRCA for New Orleans and Sydney variant were 2008.7 and 2010.7, respectively. Also, we observed a lineage of transition between New Orleans and Sydney.

**Conclusion:**

This study describes the different strains of norovirus isolated from Amazonas state in Brazil during a five-year period. Considering that NoV are capable of changing their antigenic epitopes rapidly, a continuous surveillance is important to monitor the occurrence and changes of the NoV in the community through epidemiological studies. These results contribute to the understanding of NoV molecular epidemiology and its evolutionary dynamics in Amazonas state, Brazil.

**Electronic supplementary material:**

The online version of this article (10.1186/s12879-018-3068-y) contains supplementary material, which is available to authorized users.

## Background

Norovirus (NoV) is considered a major cause of non-bacterial gastroenteritis worldwide [1]. These viruses are highly infectious, requiring a low viral load to cause infection (≥18 particles), and they are environmentally stable for long periods [2]. These characteristics increase their infectivity and facilitate their transmission and spread, which can cause outbreaks, hospitalization, and global epidemics [3].

The main transmission route of the noroviruses is fecal-oral, contact with infected persons, ingestion of contaminated water or food, or aerosols produced by vomiting [4]. A meta-analysis concluded that norovirus is responsible for 18% of the cases of acute gastroenteritis (AGE) worldwide, with approximately 24% of these cases in the community, 20% in outpatients and 17% in hospitalized patients [5].

The norovirus genome comprises single-stranded, positive sense RNA, organized into three open reading frames (ORFs). ORF 1 encodes a large polyprotein of 1738 amino acid (AA), which is cleaved by viral protease (3C) into 6 non-structural proteins, p28, NTPase, p22, VPg, 3C-like protease (3CLpro), and RNA-dependent RNA polymerase (RdRp). ORF2 encodes a major structural protein, VP1, and ORF3, a minor structural protein, VP2 [6].

The virus contains an icosahedral capsid composed of 90 dimers of VP1 protein, which consists of two domains, the shell (S) domain and protruding (P) domain [7]. The S domain (AA 50-225) is more related to the structure of the capsid. The P domain (AA 226-530) is subdivided into two subdomains, P1 and P2. The P2 subdomain contains important determinants of antigenicity, being responsible for binding to histoblood group antigens (HBGA), which function as attachment factors or co-receptors on host cells [7, 8]. Changes in the P2 nucleotide sequence of GII.4 strains are associated with the emergence of new pandemic/epidemic strains (variants) with alterations in their antigenicity profiles [8].

The genus *Norovirus* is classified into at least six genogroups (GI to GVI) [9], which are subdivided into more than 40 genotypes. Viruses from genogroups GI, GII, and GIV are known to infect humans [6]. The GII.4 is the predominant genotype responsible for the majority of norovirus outbreaks [10].

Emergence of new GII.4 variants every two to three years is associated with most norovirus pandemics. Since 1995, six GII.4 pandemic variants have emerged, which were denominated as US 95/96, Farmington Hills_2002, Hunter_2004, Den Haag_2006b, New Orleans_2009, and Sydney_2012 [11]. In addition, other GII.4 variants have been described, including Asia 2003 and Yerseke 2006a, both of which were related to additional limited outbreaks [12].

In the present study, a molecular approach was designed for a phylogenetic analysis of norovirus lineages in Amazonas state, Brazil, over five years. Sequence analysis of the polymerase, capsid, and P2 subdomain regions was successfully used for the identification of genotypes, as well as for characterization of the recombinant strains.

## Methods

### Selection of clinical specimens and norovirus detection

Totally, 1053 fecal specimens were collected from children (< 10 years old) with acute gastroenteritis symptoms by the National Program for Surveillance of Rotavirus Gastroenteritis in Manaus city, Amazonas State, between January 2010 to December 2014. The program investigated sporadic cases of diarrhea from inpatients that used public health facilities. These samples were tested for the presence of norovirus by an enzyme immunoassay (EIA) using the RIDASCREEN® Norovirus 3rd Generation EIA kit (R-Biopharm, Darmstadt, Germany) resulting in 349 positive samples distributed over the five years. Seventy one percent of these positive samples with available material (*n* = 250; 2010 = 36; 2011 = 33; 2012 = 70; 2013 = 52; 2014 = 59) were selected for genotyping and amplification by reverse transcription polymerase chain reaction (RT-PCR).

### Ethical considerations

This study was approved by the Ethics Committee on Human Research of Evandro Chagas Institute, Brazilian Ministry of Health (CEPH/IEC protocol No. 0017/2014 update No. 1.318.103 of 2015).

### Nucleic acid extraction and reverse transcription

Nucleic acids were extracted using the silica method [13]. The extracted genetic material was submitted to reverse transcription (RT) with a random primer [pd(N)^6^™ (Amersham Biosciences, UK)] using the enzyme Superscript™ II Reverse transcriptase (Invitrogen, USA).

### Norovirus RNA amplification

Norovirus-positive samples were amplified by RT-PCR, targeting the regions B of polymerase gene (213 bp) and D of the capsid (253 bp) in the viral genome, using primers Mon 431/432/433/434 [14] and Cap C/D1/D3 [15], respectively. The GII.4 strains were also amplified with the primers EVP2F/EVP2R (653 bp) [16], targeting the hypervariable capsid region, P2 subdomain. To investigate the samples with different genotypes on polymerase and capsid genes, PCR was performed, targeting the junction region of ORF1/2, using the primers Mon 431 and G2SKR [14, 17].

### DNA purification and sequencing

The amplicon was purified with the QIAquick^®^ PCR purification kit (QIAGEN®) or MEGAquick-spin™ Total Fragment DNA Purification Kit (iNtRON Biotechnology, Kyungki-Do, Korea) as described in the manufacturers’ protocol. Sequencing was performed with the Big Dye Terminator Cycle Sequencing Ready Reaction Kit (v.3.1) (Applied Biosystems, Foster City, CA, USA) using the same pair of primers from the PCR in an ABI Prism 3130 xl DNA Sequencer (Applied Biosystems, Foster City, USA) platform. All the reactions were accomplished with positive controls (positive standard sample - norovirus GII.4) and negative controls (DNase/RNase- Free Water). The sequences generated were deposited in GenBank under accession numbers MF401649-MF401943.

### Molecular and phylogenetic analyses

Preliminary analyses of the genotypes were performed in Norovirus Genotyping Tool v.1.0 (http://www.rivm.nl/norovirus/typingtool) [18]. Phylogenetic analyses were performed using Maximum-Likelihood with IQTree program v.1.3.0 [19] by Ultrafast Bootstrapping (UFboot) [20]. The statistical significance of phylogenies constructed was estimated with 1000 replicates. Edits on the phylogenetic trees were done with the program FigTree v.1.4.2 [21].

Analysis of the epitopes of the P2 region was done in the MEGA 6 program [22]. The prototype sequences were obtained from GenBank database of the National Center for Biotechnology Information (NCBI). The recombination analyses were performed using the Simplot v. 3.5.1 program [23].

### Evolutionary analysis of norovirus GII.4 variants

In order to conduct time-measured phylogenetic analysis, P2 subdomain sequences were tested by the Bayesian Markov Chain Monte Carlo (MCMC) method implemented in Bayesian Evolutionary Analysis Sampling Trees (BEAST) v1.8.2 [24]. The most recent common ancestors (TMRCA) were estimated by a relaxed clock, uncorrelated log-normal molecular clock model [25]. TMRCA was determined using the Coalescent Piecewise Bayesian Skyride Plot method [26] with 100 million replicates (more details in Additional file 1: Table S1). Nucleotide variations within and between clusters were examined by applying the maximum likelihood based on the GTR + I + G nucleotide substitution model, chosen by jModelTest v. 2.1 [27].

### Protein modeling

The 3D structure was built using protein homology modeling. The initial search and selection were done using templates from Protein Database Bank (PDB) (https://www.rcsb.org/), using the norovirus capsid. The selected templates were 1IHM and 4OP7. The MODELLER^©^ v. 9.15 software was used to build the 3D models. After protein modeling, the results were validated using the PROCHECK [28] and VERIFY3D programs [29] in order to check the biochemical parameter quality. A visual inspection was performed using the PyMOL Molecular Graphics System v. 1.8 (Schrödinger, LLC).

## Results

During the period of January 2010 to December 2014, 33,1% (349/1053) samples were positive for norovirus by EIA test. Seventy one percent (250/349) with sufficient material were tested by RT-PCR and 189 (75.6%) amplified by at least one region, and then directly sequenced (Table 1).

**Table 1:** Positivity rates obtained for norovirus in fecal samples from children with acute gastroenteritis, from Manaus, Amazonas, Brazil, between 2010 and 2014, using an enzyme immunoassay (EIA) and polymerase chain reaction (PCR)

Sample/Year	2010 Pos/Total (%)	2011 Pos/Total (%)	2012 Pos/Total (%)	2013 Pos/Total (%)	2014 Pos/Total (%)	TOTAL Pos/Total (%)
Tested by EIA	58/162 (35,8)	69/264 (26,1)	97/285 (34)	63/155 (40,6)	62/187 (33,1)	349/1053 (33,1)
Tested by PCR^a^	18/36 (50)	26/33 (78,8)	58/70 (82,9)	42/52 (80,8)	45/59 (76,3)	189/250 (75,6)

*Pos* Positive, *EIA* enzyme immunoassay, *PCR* polymerase chain reaction

^a^Positive by at least one region of the genome

**Fig. 1:**
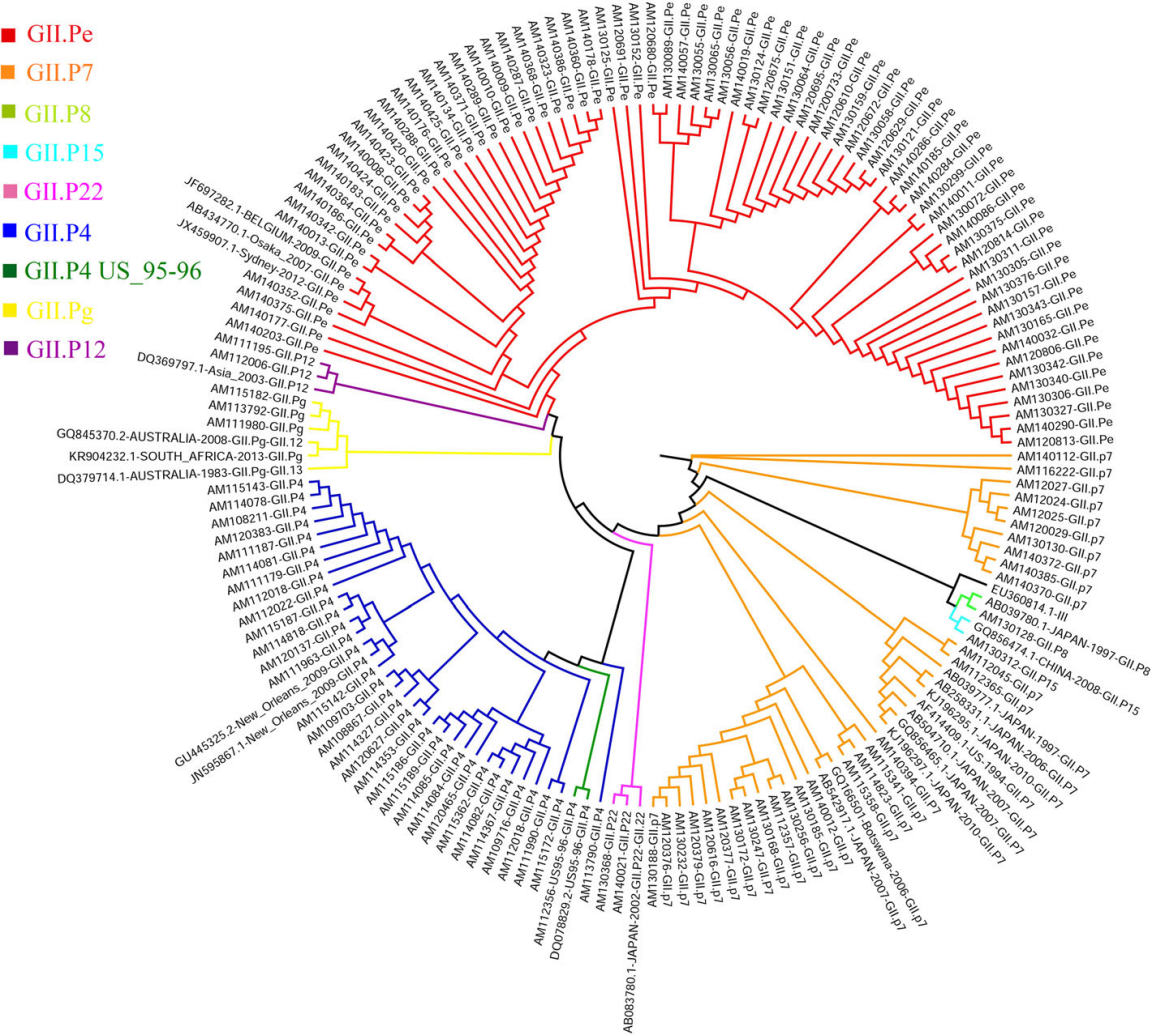
Maximum likelihood clade tree, based on the RNA polymerase gene (200 bp) of 143 partial nucleotide sequences of norovirus from children with acute gastroenteritis in Manaus city, Amazon, Brazil. The color of the branches is based on the NoV-genotype. All samples that were studied begin with the initials AM, and 35 identical sequences of the same are not shown

**Fig. 2:**
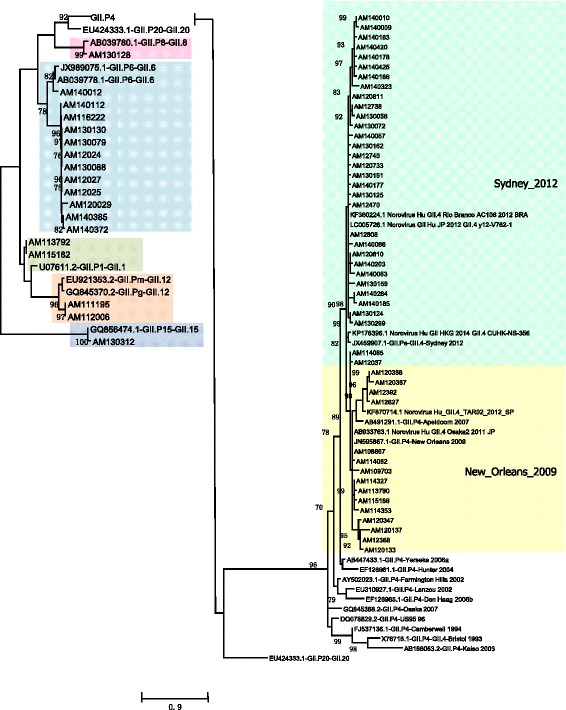
Maximum likelihood phylogenetic analysis based on the capsid gene (D region of VP1) (188 bp) of 64 partial nucleotide sequences of norovirus from children with acute gastroenteritis in Manaus city, Amazon, Brazil. The color of the branches is based on the NoV-genotype. All samples that were studied begin with the initials AM, and 67 identical sequences of the same are not shown

Based on the partial sequence of the capsid and polymerase region, the norovirus sequences were classified using the binary nomenclature as described by Kroneman et al. [30]. The GII.4 (GII.Pe/GII.4 and GII.P4/GII.4) was the genotype most frequently detected, with a prevalence of 52.4% (99/189) (Table 2), followed by GII.P7/GII.6, with 6.3% (12/189). Some strains were only sequenced by their capsid or polymerase region (Table 2). Less frequent genotypes such as GII.P8/GII.8, GII.P15/GII.15, and GI.P5/GI.5 were also found (Figs. 1 and 2). The unusual recombinant strains found in this study have already been described in a preliminary study with further details [31].

**Table 2:** Genotypes of norovirus obtained in 189 positive fecal samples from children with gastroenteritis, from Manaus, Amazonas, Brazil, between 2010 and 2014

*Pol+/Cap+*	*Nº of cases (%)*
GII.Pe/GII.4 Sydney_2012	64 (33.9)
GII.P4/GII.4 New Orleans_2009	35 (18.5)
GII.P7/GII.6	12 (6.3)
GII.P8/GII.8	1 (0.5)
GII.P15/GII.15	1 (0.5)
GII.P22/GII.5	1 (0.5)
GII.P12/GII.12	2 (1.1)
GII.Pg/GII.1	2 (1.1)
GII.Pg/GII.12	1 (0.5)
GI.P5/GI.5	1 (0.5)
*Pol+/Cap-*	
GII.Pe/NA	26 (13,8)
GII.P4/NA	13 (6,9)
GII.P7/NA	17 (9,0)
GII.P4_US95_96/NA	1 (0,5)
GII.P22/NA	1 (0,5)
*Pol-/Cap+*	
NA/GII.4	9 (4,8)
NA/GII.6	2 (1,1)

*NA* not assigned

### Norovirus GII.4 variants

**Fig. 3:**
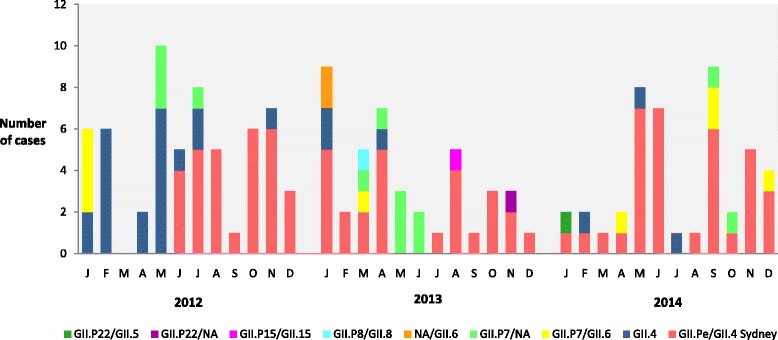
Monthly distribution of NoV-genotypes associated with acute gastroenteritis in Manaus city, Amazon, Brazil, between 2012 and 2014

Between the years 2010 and 2011, we detected the GII.P4/GII.4 New Orleans_2009 strain at higher frequency, mainly in the year 2011 (data not shown). The epidemiological data obtained in this period was already described by Costa et al. [32]. Thus, the temporal distribution showed in this present study only involved the period from 2012 to 2014. The temporal genotypic distribution demonstrated the emergence and spread of a Sydney_2012 variant from June 2012 until 2014, replacing the New Orleans_2009 strain (Fig. 3). During this period, GII.Pe also circulated; this is a signatory strain of the GII.4 Sydney-2012 in samples genotyped only for the polymerase gene. The recombinant strain GII.P7/GII.6 was the second most frequent, observed in samples from all the years studied, except 2010 (Fig. 3).

**Fig. 4:**
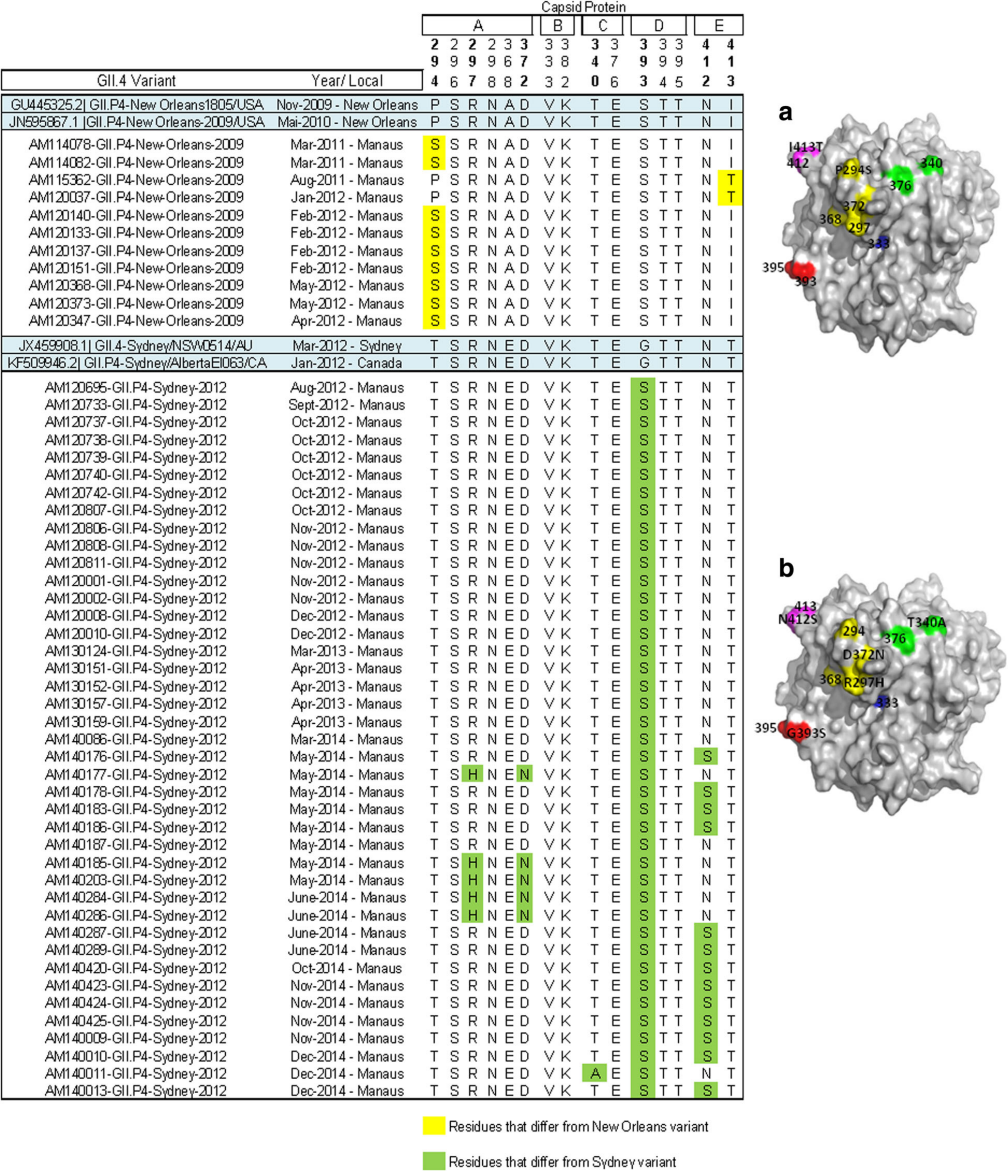
Alignment of antigenic residues of capsid protein (P2 region) in strains circulating in Brazil, 2011-2014. Antigenic residues are divided in five epitopes (A-E). The prototype strains representative of the epidemic strain variants that emerged between 2009 and 2012 are highlighted in blue. Amino acid residues that differ from those of the prototype are highlighted in yellow and green. **a** Structure modeling of the VP1 protein (PDB 4OP7) showing the amino acid replacement in NoV GII.4 New Orleans_2009 lineages from Manaus, Brazil. Antigenic epitopes are colored in yellow (A), blue (B), green (C), red (D), and purple (E). **b** Structure modeling of the VP1 protein (PDB 4OP7) showing the amino acid replacement in NoV GII.4 Sydney_2012 lineages from Manaus, Brazil

Amino acid analysis of the P2 subdomain of GII.4 norovirus showed non-synonymous nucleotide mutations for the two circulating GII.4 strains (Fig. 4). In the New Orleans_2009 strains, two of these mutations occurred in putative A (AA 294) and E epitopes (AA 413) (Fig. 4). Both changes modified the chemical nature of the AA from apolar to polar uncharged. Other changes outside of this epitope were identified, including the AA 341 in 75% of the strains New Orleans_2009 obtained in 2012 (data not shown), but its importance is not well established.

The GII.4 Sydney 2012 variant had an accumulation of several changes in putative epitopes on the P2 subdomain. Thus, when Sydney_2012 strains were analyzed we identified five changes in AA antigenic epitopes: 297 and 372 (A epitope), 340 (C epitope), 393 (D epitope) and 412 (E epitope) (Fig. 4). The majority (4/5) of these changes occurred in strains collected in 2014, except for the alteration of AA 393 (D epitope) that was present in 97% of the total samples. Three of these changes modified the chemical nature of the AA: Polar uncharged for Apolar (340) from negative to Polar uncharged (372), Apolar for Polar non-loaded (393).

**Fig. 5:**
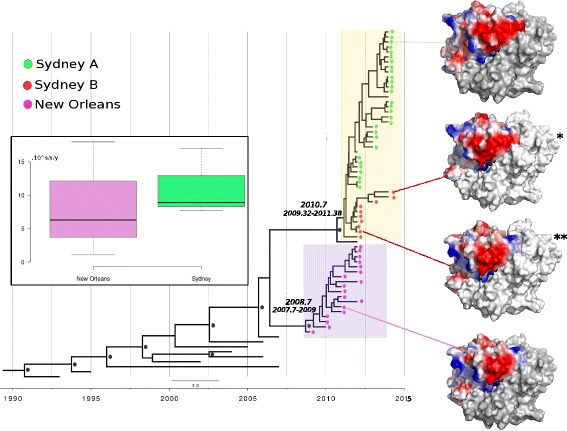
Molecular clock phylogeny based on the 58 nucleotide sequence (627 bp) of P2 region (VP1), estimated by uncorrelated log-normal model using the Coalescent Piecewise Bayesian Skyride Plot method with 100 million replicates, from 2010 to 2014 in Amazon, Brazil. The taxa were represented by colored circles according to the GII.4 variant. The most recent common ancestor (TMRCA) of each variant is indicated next to the clade. The black circle represents supported clades (> 95%). Three-dimensional VP1 structure of the GII.4 predicted by homologous modeling based on the crystal structure (PDB number 4OP7) (*) Surface-exposed electrostatic charges showing differences within the Sydney lineage. (**) Surface-exposed electrostatic charges showing a sample clustering with Sydney variant but demonstrating more similarities in the surface protein with New Orleans variant. Boxplot of evolutionary rates of Sydney and New Orleans variants

Structural analysis of the protein demonstrated several changes in the distribution of the surface electrostatic charges (Fig. 5). We note that the Sydney_2012 lineage is divided into two clusters (named Sydney A and B). The Sydney_B lineage seems to be a transition between the New_Orleans_2009 and Sydney_2012 variants. More phylogenetically close samples to the New Orleans lineage showed high similarity in the protein surface with the New Orleans strain; likewise, samples close to the Sydney_A variant were similar to the Sydney_2012 strain, indicating that the division of the clades within the Sydney_2012 samples is associated with amino acids and not only with nucleotide changes.

To investigate the temporal evolutionary dynamics of the GII.4 norovirus in the Amazon, we applied Bayesian coalescent analysis in 58 P2 (VP1) sequences from 2010 to 2014, implemented in the BEAST package. Different population dynamics models were tested (constant, exponential, expansion, skyline) and the better values of ESS (effective sample size) revealed that the skyride population growth model was the best fit to the data. The uncorrelated log-normal model estimations calculated higher rate of evolution, 7.7 × 10^− 3^ for the Sydney variant and 6.3 × 10^− 3^ for New Orleans. The same Bayesian inference estimated the time to TMRCA. The year of TMRCA from the population analyzed was 2008.7 to New Orleans strains and 2010.7 to Sydney. The boxplot showed that the variation in the evolutionary rate was higher in the Sydney (7.7 × 10^− 3^ average) strains, but the interval was larger than for New Orleans (1.1 × 10^− 3^ to 1.8 × 10^− 2^) (Fig. 5).

## Discussion

Considering the high genetic diversity and elevated rates of evolution of norovirus, continuous surveillance of cases for monitoring genotypes and the emergence of new strains is required. The evolution of the GII.4 pandemic strains is a consequence of point mutations in the P domain capsid, and genomic recombination events between ORF1 and ORF2 [33].

In epidemiological investigations carried out in several countries, the GII.4 strain was observed as the cause of most cases, corroborating with the findings obtained in this study [12, 34]. The pattern observed in the variants of GII.4 in Manaus was similar to that found across the world, where new variants appear every 2 or 3 years, replacing the previous one [35]. Between 2009 and 2012, the New Orleans pandemic strain was responsible for 75% of the outbreaks of diarrhea in New Zealand and Australia [36]. In some studies, it was possible to observe its co-circulation with other variants of GII.4, such as Den Haag_2006b [10, 37]. This co-circulation was found among the analyzed samples of Manaus since 2012, where Sydney_2012 was predominant and New Orleans_2009 appeared in few cases.

It is known that recombination events are frequently evolutionary mechanisms in the genomes of norovirus and that they can strongly influence phylogenetic grouping [38]. The GII.Pg/GII.12 strain found in Manaus in February 2010 shares 99.8% nucleotide identity with other recombinants that circulated in Rio Grande do Sul (South of Brazil) in 2009 (KR074161-62, KR074190-91), indicating a possible circulation in the country. Moreover, data from the literature report that this recombinant has emerged on almost all continents between the years 2009 and 2011 [39–42].

Interestingly in this work, the recombinant strain GII.P7/GII.6 was detected in all years between 2011 and 2014. Recombinant strains are usually found in few cases or causing outbreaks [43]. This fact suggests that this lineage is well established in the population and that more comprehensive studies involving immunogenicity would be necessary to assess its impact. The genotypes GII.6 and GII.7 are frequently described in several studies conducted in Brazil, often behind only GII.4 strains [44, 45]. In most epidemiological studies, genotyping was performed by sequencing only one region of the genome, the viral capsid, which makes it difficult to understand the actual epidemiology of the recombinant strains. The data obtained in the present study reinforce the need for binary classification of norovirus genotypes, as already suggested by Kroneman et al. [30].

By the analysis performed on the subdomain P2, changes were found in both the GII.4 New Orleans_2009 and the Sydney_2012 strains. It is known that this subdomain interacts with neutralizing or blocking antibodies, and with HBGA ligands. Several studies using human and mouse monoclonal antibodies against norovirus VLPs in neutralization assays have identified a correlation between the emergence of novel GII.4 epidemic strains and amino acid changes in specific epitopes (A-E). Such changes alter the immunogenicity pattern, resulting in an escape from the action of the immune system [46].

The alterations found in the Manaus strains indicate that residues 294/413 and 297/372 were identified as antigenic determinants of New Orleans and Sydney 2012, respectively. Thereby, the New Orleans and Sydney lineages have undergone significant mutations in blocking epitopes, and already exhibit differences with respect to the prototypes. In the Sydney strains, it is possible to observe that the changes in epitopes occurred only from May 2014, which may indicate that over the years 2012 and 2013, these strains were acquiring point mutations, culminating in amino acid changes in the strains from 2014.

It is known that RNA viruses have a high evolution rate, higher than the DNA genomes, mainly because they do not have repair mechanisms for their replication [47]. The relaxed-clock estimations calculated similar rates of evolution in Manaus (Sydney: 7.7 × 10^− 3^; New Orleans: 6.3 × 10^− 3^) to other studies, which reported 6.99 × 10^− 3^ and 7.3 × 10^− 3^ subst./site/year for full VP1 and subdomain P2 gene sequences, respectively [48, 49]. In a study conducted in Belem, a state adjacent to Manaus, over 30 years, Siqueira et al. [50] found an evolutionary rate of 9.05 × 10^− 3^ subs./site/year for other GII.4 variants. The time-scale evolutional was constructed based on the subdomain P2, which is the most hypervariable region in VP1, which are under selection pressure of the immunological system. Its can explain the reasons to the high evolutionary rates founded in this study. Further studies should be carried out for studying the time-scale evolutionary phylogeny and phylodynamics of the VP1 and RdRp genes.

The amino acid changes acquired over the years in samples from the present study are already reflected in the 3D structure of the protein, which may have allowed the virus to evade host immune surveillance. Recently, the emergence of the GII.17_Kawasaki strain has been observed in several countries, but it is important to note that GII.4 strains still play a key role in norovirus cases.

One of the limitations that may be considered in this study is the use of EIA for norovirus screening, taking into account the limited detection potential of this technique over the current gold standard quantitative PCR (qPCR), as well as lower sensitivity against emerging genotypes. However, a research conducted by Siqueira et al. [51] demonstrated a good performance using the same EIA kit, with a sensitivity of 92% and a specificity of 83.3%. This methodology has already been used in other studies, carried out in the Amazon region, establishing higher positive rates [44, 52, 53]. Uncommon recombinants strains (GII.P21/GII.2, GII.13/GII.17, GII.P21/GII.3) and emergent variants (GII.4_Sydney) were also detected by EIA [50, 54].

## Conclusion

The research conducted in the present study was based on the epidemiological and molecular surveillance of norovirus strains, in samples collected from an important state of the Brazilian Amazon region, over a period of five years. Our data indicate that noroviruses are an important cause of gastroenteritis in the Amazon region. Although highly diverse, NoV circulating over the past 5 years was predominantly characterized as GII.4, including GII.4 variants New Orleans_2009 and Sydney_2012. Monitoring of GA cases caused by norovirus is essential to evaluate the impact of this virus in the community (sporadic cases and outbreaks), as well as for the development and evaluation of control measures, such as vaccines.

## Additional file

Additional file 1: **Table S1.** Nucleotide substitution rate and divergent times using 10 million generations. (DOC 43 kb)

## Abbreviations

3CLpro: 3C-like protease
AA: Amino acid
AGE: Acute gastroenteritis
BEAST: Bayesian Evolutionary Analysis Sampling Trees
EIA: Enzyme immunoassay
ESS: Effective sample size
HBGA: Histoblood group antigens
MCMC: Bayesian Markov Chain Monte Carlo
NoV: Norovirus
ORFs: Open reading frames
P: Protruding
PDB: Protein Database Bank
qPCR: Quantitative polymerase chain reaction
RdRp: RNA-dependent RNA polymerase
RT: Reverse transcription
RT-PCR: Reverse transcription polymerase chain reaction
S: Shell
TMRCA: Most recent common ancestors
