# Supplementary material for: Molecular epidemiology and temporal evolution of norovirus associated with acute gastroenteritis in Amazonas state, Brazil
Source: BMC Infect Dis. 2018 Apr 2;18:147. doi: 10.1186/s12879-018-3068-y (PMC5879549; doi:10.1186/s12879-018-3068-y)
Supplement: Supplementary file 1 — Table S1. Nucleotide substitution rate and divergent times using 10 million generations. (DOC 43 kb) [file 12879_2018_3068_MOESM1_ESM.doc]

| **Molecular clock** | **AICM** | **S.E.** | **Nucleotide substitution rate(10^-3^ rate/site/year)** | **TMRCA by:** | |
| --- | --- | --- | --- | --- | --- |
|  |  |  |  | **No. of years** | **Date (range)** |
| Exponential relaxed clock | 11129,358 | +/- 5.843 | 5.8 (2.92-7.55) | 21.4 (12.4-35.3) | 1992.2 (1978.7-2001.6) |
| Lognormal relaxed clock | 10174,075 | +/- 0.586 | 4.97 (3.54-6.5) | 22.7 (14.33-33.7) | 1991.3 (1980.3-1999.67) |
| Strict clock | 10215,035 | +/- 0.654 | 4.79 (4.12-5.51) | 21.75 (18.35-25.15) | 1992.25 (1988.85-1995.65) |
| Lognormal Bayesian Skyline | 20037,621 | +/- 34.306 | 4.87 (1.48-6.12) | 22.2 (12.6-47.8) | 1991.8 (1966.2-2001.4) |
| Lognormal constant size | 27634,236 | +/- 81.923 | 4.35 (0.62-5.8) | 35.7 (15.31-129.65) | 1978.3 (1884.35-1998.69) |
| Lognormal exponential growth | 10193,756 | +/- 2.528 | 4.98 (3.68-6.51) | 22.04 (15.49-30.13) | 1991.96 (1952.9-1998.5) |
| Lognormal expansion growth | 10955,951 | +/- 7.821 | 4.46 (1.0-6.25) | 28.04 (15.5-61.1) | 1985.96 (1952.9-1998.5) |
| Lognormal extended skyline plot | 14701,635 | +/- 33.792 | 4.52 (1.0-6.03) | 26.08 (13.24-65.99) | 1987.92 (1948.01-2000.76) |
| Lognormal logist growth | 11026,857 | +/- 4.517 | 4.97 (1.96-6.45) | 21.41 (14.05-37.60) | 1992.59 (1976.4-1999.95) |
| Lognormal Bayesian Skygride | 10034,285 | +/- 0.853 | 4.39 (1.0-5.83) | 24.90 (12.70-60.28) | 1989.1 (1953.8-2001.3) |
| Lognormal GMRF Bayesian Skyride | 9982,353 | +/- 2.119 | 4.08 (0.66-6.48) | 19.79 (11.05-49.29) | 1994.21 (1964.71-2002.95) |

Table 1 - Nucleotide substitution rate and divergent times using 10 million generations.
